# N-Terminal pro C-Type Natriuretic Peptide (NTproCNP) and myocardial function in ageing

**DOI:** 10.1371/journal.pone.0209517

**Published:** 2018-12-19

**Authors:** Bryan M. H. Keng, Fei Gao, Ru San Tan, See Hooi Ewe, Louis L. Y. Teo, Bei Qi Xie, George B. B. Goh, Woon-Puay Koh, Angela S. Koh

**Affiliations:** 1 National Heart Centre Singapore, Singapore, Singapore; 2 Duke-NUS Medical School, Singapore, Singapore; 3 Singapore General Hospital, Singapore, Singapore; 4 Saw Swee Hock School of Public Health, National University of Singapore, Singapore, Singapore; Scuola Superiore Sant'Anna, ITALY

## Abstract

Ageing-related alterations in cardiovascular structure and function are commonly associated with chronic inflammation. A potential blood-based biomarker indicative of a chronic inflammatory state is N-Terminal Pro C-Type Natriuretic Peptide (NTproCNP). We aim to investigate associations between NTproCNP and ageing-related impairments in cardiovascular function. Community-based participants underwent same-day assessment of cardiovascular function and circulating profiles of plasma NTproCNP. Associations between cardiovascular and biomarker profiles were studied in adjusted models including standard covariates. We studied 93 participants (mean age 73 ± 5.3 years, 36 women), of whom 55 (59%) had impaired myocardial relaxation (ratio of peak velocity flow in early diastole E (m/s) to peak velocity flow in late diastole by atrial contraction A (m/s) <0.84). Participants with impaired myocardial relaxation were also found to have lower peak early phase filling velocity (0.6 ± 0.1 vs 0.7 ± 0.1, p < 0.0001) and higher peak atrial phase filling velocity (0.9 ± 0.1 vs 0.7 ± 0.1, p < 0.0001). NTproCNP levelswere significantly lower among participants with impaired myocardial relaxation (16.4% vs 39.5% with NTproCNP ≥ 19, p = 0.012). After multivariable adjustments, NTproCNP was independently associated with impaired myocardial relaxation (OR 2.99, 95%CI 1.12–8.01, p = 0.029). Community elderly adults with myocardial ageing have lower NTproCNP levels compared to those with preserved myocardial function. Given that impaired myocardial relaxation probably represents early changes within the myocardium with ageing, NTproCNP may be useful as an ‘upstream’ biomarker useful for charting myocardial ageing.

## Introduction

Current knowledge suggests that ageing-related alterations in cardiovascular structure and function are commonly associated with chronic inflammation mediated by various factors[1]. Proinflammatory mechanisms cause age-associated arterial remodelling and impaired myocardial relaxation at the onset of diastole, reducing the early left ventricular filling rate[2,3]. Despite this knowledge, measures that specifically tackle these deleterious alterations in cardiovascular structure and function associated with chronological ageing are sorely lacking. For instance, hardly any biomarkers have been specifically studied as candidate biomarker for cardiovascular ageing.

The natriuretic peptides (NPs) represent a family of structurally related but genetically distinct peptide hormones that include atrial NP (ANP), brain/B-type NP (BNP) and C-type NP (CNP)[4]. ANP and BNP are synthesized largely in the cardiac chambers and are released into the circulation in response to wall stretch such as in heart failure states, exerting direct natriuretic function [4–6]. BNP is deglycosylated from a 108-amino acid prohormone, proBNP, and further processed into an amino terminal fragment, NTproBNP and a biologically active BNP peptides [7]. Unlike ANP and BNP, CNP is highly expressed in the brain and also present in particularly high concentrations in the vascular endothelium, inducing vasorelaxation and vascular remodelling[8]. NTproCNP is produced by cleavage of a prohormone with 103 amino acids, proCNP, yielding the amino terminal fragment (NTproCNP) and biologically active CNP peptides [9]. CNP does not have direct natriuretic activity, but is instead an endothelium-derived peptide which protects the endothelium in response to cardiovascular injury and disease[10–12]. Natriuretic peptide receptors for ANP and BNP are guanylyl cyclase-linked, utilizing cyclic guanosine monophosphate as the intracellular messenger. On the other hand, natriuretic peptide receptor for CNP is not linked to guanylyl cyclase, but is involved in clearance of CNP from the circulation [13–15].

CNP is released in response to pro-inflammatory cytokines, possibly involving interactions between macrophageal cytokines and vascular endothelium[16,17]. This link may indicate a potential role of CNP in cardiovascular ageing, which is also characterized by proinflammatory alterations in arterial and myocardial remodelling. Therefore, CNP may be a potential blood-based biomarker representative of chronic inflammation with ageing.

NTproCNP has a longer half-life and is present in the blood at higher concentrations than biologically active CNP. As such, NTproCNP may in fact demonstrate even more consistent data than CNP in terms of clinical application [18]. Furthermore, levels of NTproCNP also show significant correlation with CNP concentrations, as both molecules are released from cells at an equimolar ratio[19]. This makes NTproCNP a clinically useful proxy for measuring CNP levels.

In this study, we hypothesise that NTproCNP, being closely related to the inflammatory processes present in ageing, may show correlation with impairments in myocardial relaxation, in an elderly community-based population, prior to development of cardiovascular disease.

## Materials and methods

The subjects were recruited from the Cardiac Ageing Study (CAS)[20], a prospective study initiated in 2014 that examines characteristics and determinants of cardiovascular function in elderly adults. CAS participants were recruited from the local community, and also from the prospective, population-based cohort, The Singapore Chinese Health Study (SCHS)[21].

The study sample consisted of men and women who were recruited between 2014 and 2017 and who had no self-reported history of physician-diagnosed cardiovascular disease (such as coronary heart disease, stroke) or cancer. Written informed consent was obtained from participants upon enrolment. The SingHealth Centralised Institutional Review Board (2014/628/C) had approved the study protocol. All methods were performed in accordance with the relevant guidelines and regulations.

As previously described [20,22], all participants were examined and interviewed on one study visit by trained study coordinators. Participants completed a standardized questionnaire that included medical history and coronary risk factors. Hypertension was defined by current use of antihypertensive drugs or physician-diagnosed hypertension. Diabetes mellitus was defined by current use of anti-diabetic agents or physician-diagnosed diabetes mellitus. Dyslipidaemia was defined by current use of lipid-lowering agents or physician-diagnosed dyslipidaemia. Smoking history was defined as ever smokers (former or current smoking) or never smokers. Body mass index was calculated as weight in kilograms divided by the square of height in meters. Sinus rhythm status was ascertained by resting electrocardiogram. Clinical data were obtained on the same day as assessment of echocardiography and serum collection.

### Transthoracic echocardiography imaging

We performed standard echocardiography for each subject using ALOKA α10 with a 3.5 MHz probe. Standard echocardiography that involved 2-D, M-mode, pulse Doppler and tissue Doppler imaging, was performed in the standard parasternal and apical (apical 4-chamber, apical 2-chamber and apical long) views, and three cardiac cycles were recorded. We measured left ventricular ejection fraction (LVEF), left atrial (LA) volume and LA volume index. Using Doppler echocardiography, trans-mitral flow E and A wave with the sample volume position at the tip of the mitral valve leaflets from the apical 4-chamber view were recorded. E/A ratio was computed as a ratio of peak velocity flow in early diastole E (m/s) to peak velocity flow in late diastole by atrial contraction A (m/s). From the apical 4-chamber view, pulsed wave tissue Doppler imaging was performed with the sample volume at the septal and lateral annulus. The frame rate was between 80 and 100 frames per second. All measurements were measured by the same operator and the measurements were averaged over three cardiac cycles and adjusted by the RR interval.

### Laboratory assessments

Plasma levels of NTproCNP were measured using the NT-proCNP ELISA kit (DRG Instruments GmbH Germany), according to the manufacturer’s assay procedures. Briefly, 50 μL of assay buffer was added into each well, followed by 20 μL of plasma samples and 50 μL of conjugate. The plate was sealed and incubated at room temperature for three hours. Subsequently, the plate was washed five times before 100 μL of substrate was added to each well, then incubated for another thirty minutes at room temperature in the dark. 50 μL of stop solution was added and read at 450 nm immediately. Plasma levels of NTproBNP were measured with NTproBNP assay (proBNP II, code 04842464 190, Roche Diagnostics, Germany) using monoclonal electrochemiluminescence immunoassay (ECLIA) method.

### Statistical methodology

We first examined bivariable association of subject clinical characteristics, cardiac function and biomarkers with impaired myocardial relaxation. Impaired myocardial relaxation was defined as ratio of peak velocity flow in early diastole E (m/s) to peak velocity flow in late diastole by atrial contraction A (m/s) less than 0.84. (mean E/A ratio was 0.84 in our study sample).

The t test and chi-square test were used for continuous variables and categorical variables respectively. Continuous variables are reported as a mean with standard deviation (SD).

Multivariable logistic regression models were constructed to assess the association of NTproCNP with impaired myocardial relaxation. The initial univariate logistic regression model examined the individual association with demographic variables and clinical covariates. Those variables associated in the univariate analysis with a p < 0.05 were candidate NTproCNP effect modifiers. The potential effects of these candidates were then assessed individually via multivariable logistic regression.

All statistical analyses were performed using STATA 13 (College Station, Texas, USA). For all analysis, a two-tailed *P* value of <0.05 was considered significant.

## Results

A total of 93 participants (mean age 73 ± 5.3 years, 36 women) were included in the analysis. Of these 93 participants, 55 (59%) had impaired myocardial relaxation (ratio of peak velocity flow in early diastole E (m/s) to peak velocity flow in late diastole by atrial contraction A (m/s) <0.84). Participants with impaired myocardial relaxation did not have other features of diastolic dysfunction: mean ratio of peak velocity flow in early diastole to peak early diastolic septal mitral annular velocity was 9.92 ± 2.3, mean peak early diastolic septal mitral annular velocity 0.06 ± 0.01, mean pulmonary artery systolic pressure 27.1 ± 7.4 mmHg and mean left atrial volume index 23.0 ± 7.0 (ml/m^2^). Notably, NTproBNP levels were low in the entire cohort (median 80 pg/ml interquartile range 45–150), with no differences in NTproBNP levels between those with impaired myocardial relaxation and those with preserved myocardial relaxation (median 74 vs 84 pg/ml) (Table 1).

**Table 1:** Baseline characteristics of study participants.

	Impaired myocardial function: E/A<0.84 (n = 55)	Preserved myocardial function: E/A≥0.84 (n = 38)	Total (n = 93)	P-value
Age (years)	73 ± 4.7	72 ± 6.1	73 ± 5.3	0.61
Female gender	26 (47.3%)	10 (26.3%)	36 (38.7%)	0.041
Body mass index (kg/m^2^)	24 (2.9)	23 (3.0)	23 (3.0)	0.24
Ever smoker	14 (25.5%)	5 (13.5%)	19 (20.7%)	0.17
Hypertension	35 (63.6%)	24 (63.2%)	59 (63.4%)	0.96
Dyslipidemia	31 (56.4%)	22 (57.9%)	53 (57.0%)	0.88
Diabetes mellitus	17 (30.9%)	6 (15.8%)	23 (24.7%)	0.097
Systolic blood pressure (mmHg)	156 (44.0)	149 (15.3)	153 (35.3)	0.35
Diastolic blood pressure (mmHg)	74 (11.8)	77 (12.5)	75 (12.1)	0.28
Pulse (beats per minute)	76 ± 15.9	71 ± 11.8	74 ± 14.5	0.091
NTproCNP≥19 (pmol/L)	9 (16.4%)	15 (39.5%)	24 (25.8%)	0.012
NTproBNP (pg/ml)				
Mean	108 ± 97.0	155 ± 273.0	127 ± 189.9	0.24
Median	74	84	80	
Interquartile rage	45–152	42–134	45–150	
Interventricular septum thickness at end diastole (IVSD) (cm)	0.8 ± 0.2	0.8 ± 0.2	0.8 ± 0.2	0.89
Interventricular septum thickness at end systole (IVSS) (cm)	1.3 ± 0.2	1.2 ± 0.2	1.3 ± 0.2	0.88
Left ventricular internal diameter end diastole (LVIDD) (cm)	4.3 ± 0.8	4.5 ± 0.5	4.4 ± 0.7	0.46
Left ventricular internal diameter end systole (LVIDS) (cm)	2.5 ± 0.5	2.5 ± 0.4	2.5 ± 0.4	0.73
Left ventricular posterior wall end diastole (LVPWD) (cm)	0.7 ± 0.1	0.7 ± 0.1	0.7 ± 0.1	0.75
Left ventricular posterior wall end systole (LVPWS) (cm)	1.4 ± 0.3	1.5 ± 0.2	1.4 ± 0.3	0.20
Left ventricular outflow tract (LVOT) (cm)	2.1 ± 0.2	2.1 ± 0.2	2.1 ± 0.2	0.66
Aortic diameter (AO) (cm)	3.2 ± 0.4	3.1 ± 0.4	3.2 ± 0.4	0.22
Left atrium (LA) (cm)	3.7 ± 0.6	3.6 ± 0.5	3.7 ± 0.6	0.48
Left ventricular ejection fraction (LVEF) (%)	73.7 ± 7.9	76.0 ± 5.5	74.6 ± 7.2	0.17
Left ventricular fractional shortening (LVFS) (%)	43.8 ± 6.6	44.8 ± 5.4	44.2 ± 6.1	0.49
Left ventricular mass index (grams/m^2^)	75.2 ± 16.6	73.0 ± 18.0	74.3 ± 17.0	0.61
Left atrial volume index (ml/m^2^)	23.0 ± 7.0	24.5 ± 9.6	23.6 ± 8.1	0.41
Isovolumic relaxation time (IVRT) (ms)	108.1 ± 25.0	95.8 ± 18.0	103.9 ± 23.4	0.087
Peak velocity flow in early diastole E (MV E peak) (m/s)	0.6 ± 0.1	0.7 ± 0.1	0.6 ± 0.1	<0.0001
Peak velocity flow in late diastole by atrial contraction A (MV A peak) (m/s)	0.9 ± 0.1	0.7 ± 0.1	0.8 ± 0.2	<0.0001
Mitral valve flow deceleration time (MV DT) (m/s)	217.7 ± 40.5	200.6 ± 36.3	210.6 ± 39.5	0.053
Pulmonary artery systolic pressure (PASP) (mmHg)	27.1 ± 7.4	28.4 ± 5.4	27.5 ± 6.8	0.45
Peak systolic septal mitral annular velocity (Septal S′) (m/s)	0.07 ± 0.01	0.08 ± 0.02	0.08 ± 0.01	0.11
Peak early diastolic septal mitral annular velocity (Septal E’) (m/s)	0.06 ± 0.01	0.08 ± 0.02	0.07 ± 0.02	<0.0001
Septal mitral annular velocity during atrial contraction (Septal A’) (m/s)	0.10 ± 0.01	0.11 ± 0.02	0.10 ± 0.02	0.29
Peak systolic lateral mitral annular velocity (m/s)	0.09 ± 0.02	0.09 ± 0.03	0.09 ± 0.02	0.33
Peak early diastolic lateral mitral annular velocity (m/s)	0.08 ± 0.02	0.10 ± 0.02	0.09 ± 0.02	0.0007
Lateral mitral annular velocity during atrial contraction (m/s)	0.12 ± 0.02	0.10 ± 0.02	0.11 ± 0.02	0.0006
Ratio of Peak velocity flow in early diastole E (MV E peak) to Peak early diastolic septal mitral annular velocity (Septal E’)	9.92 ± 2.3	10.1 ± 4.4	10.0 ± 3.1	0.75

Continuous data are shown as mean ± SD.

We studied the sensitivity and specificity of NTproCNP for determining impaired myocardial relaxation. NTproCNP value of ≥19 pmol/L was associated with impaired myocardial relaxation (84% specificity, 40% sensitivity).

The baseline characteristics of the study sample are shown in Table 1. Participants with impaired myocardial relaxation were more likely to be female (47.3% vs 26.3% were female, p = 0.041). Participants with impaired myocardial relaxation were also found to have lower peak early phase filling velocity (0.6 ± 0.1 vs 0.7 ± 0.1, p < 0.0001) and higher peak atrial phase filling velocity (0.9 ± 0.1 vs 0.7 ± 0.1, p < 0.0001). NTproCNP levels were significantly lower among participants with impaired myocardial relaxation (16.4% vs 39.5% with NTproCNP ≥ 19, p = 0.012). NTproBNP levels were similar in participants with or without impaired myocardial relaxation.

In a multivariable regression model adjusting for age, body mass index and NTproBNP, NTproCNP was associated with female gender (β -3.00, 95%CI -5.378–0.6301, p = 0.014).

At the univariate level, female gender (OR 0.40, 95%CI 0.16–0.98, p = 0.044) and NTproCNP levels ≥ 19 (OR 3.33, 95%CI 1.27–8.76, p = 0.015) were associated with impaired myocardial relaxation as denoted by the E/A ratio. Participants with impaired myocardial function (E/A ratio < 0.84) were significantly more likely to be female and less likely to have a high NTproCNP. After multivariable adjustments, NTproCNP remained independently associated with impaired myocardial relaxation (OR 2.99, 95%CI 1.12–8.01, p = 0.029). These results are presented in Table 2.

**Table 2:** Univariate and multivariate association with impaired myocardial relaxation.

	Univariate		Multivariate	
	Unadjusted OR (95% CI)	P-value	Adjusted OR (95% CI) adjusted for gender	P-value
NTproCNP	3.33 (1.27–8.76)	0.015	2.99 (1.12–8.01)	0.029
Age (years)	0.98 (0.90–1.06)	0.61	-	-
Female gender	0.40 (0.16–0.98)	0.044	0.45 (0.18–1.13)	0.091
BMI	0.91 (0.78–1.06)	0.24	-	-
Ever smoker	0.46 (0.15–1.40)	0.17	-	-
Hypertension	0.98 (0.42–2.31)	0.96	-	-
Dyslipidemia	1.06 (0.46–2.46)	0.88	-	-
Diabetes mellitus	0.42 (0.15–1.19)	0.10	-	-
Pulse	0.97 (0.95–1.004)	0.094	-	-
NTproBNP≥300	1.5 (0.35–6.41)	0.58	-	-

## Discussion

In this analysis, we evaluated the utility of NTproCNP as a biomarker of myocardial function in aged community adults. Among community adults, NTproCNP was independently associated with impaired myocardial relaxation, a common manifestation of myocardial ageing in ageing adults[23]. With ageing, left ventricular filling tends to decrease in early diastole, reducing the mitral ratio of peak early to late diastolic filling velocity.

This community cohort of elderly adults did not have clinical cardiovascular disease, such as heart failure, as suggested by relatively low NTproBNP levels. These low levels of NTproBNP are below suggested cut points for excluding asymptomatic heart failure in elderly adults, of particular relevance to our study[24]. Therefore, the myocardial alterations seen in this asymptomatic study population represent changes related to ageing processes.

Age-related diastolic dysfunction has been attributed to an increased passive stiffness, which is associated with increases in left ventricular fibrosis[25]. Lower NTproCNP levels were associated with worsening early to late diastolic filling velocity in our study. This is in agreement with observations in ageing rodents which reported reciprocal increases in left ventricular fibrosis associated with declines in circulating CNP levels[26].

Currently, there are limited studies on NTproCNP in cardiovascular disease in pre-specified cohorts. Our study advances current understanding on NTproCNP as a potential biomarker for cardiovascular states. To the best of our knowledge, we are the first to show a direct association between NTproCNP and measures of myocardial function in community elderly adults. Existing studies exploring NTproCNP as a novel biomarker have largely focused their attention on cohorts with clinical cardiovascular disease [27–29], with hardly any evidence available in relatively healthy human beings. Our results show promise for using NTproCNP in non-disease cohorts, such as in community based asymptomatic adults. Specifically, our results contribute to the current lack of tools available in the investigation of cardiac ageing processes in the humans, and introduce the potential to use NTproCNP as a biomarker of cardiac ageing in human cohorts.

Notably, despite the well-established role of NTproBNP as a diagnostic and prognostic biomarker of heart disease such as heart failure[30], we did not observe associations between myocardial function and NTproBNP in our study sample. NTproBNP levels were similar among those with or without impaired myocardial relaxation in our study. This reinforces the critical need for alternative biomarkers, rather than biomarkers established for clinical disease, to antedate processes prior to expression of clinical disease. Given that impaired myocardial relaxation probably represents early changes within the myocardium with ageing, its distinct association with NTproCNP suggests that NTproCNP may be useful as an ‘upstream’ biomarker useful for charting myocardial ageing.

**Fig 1:**
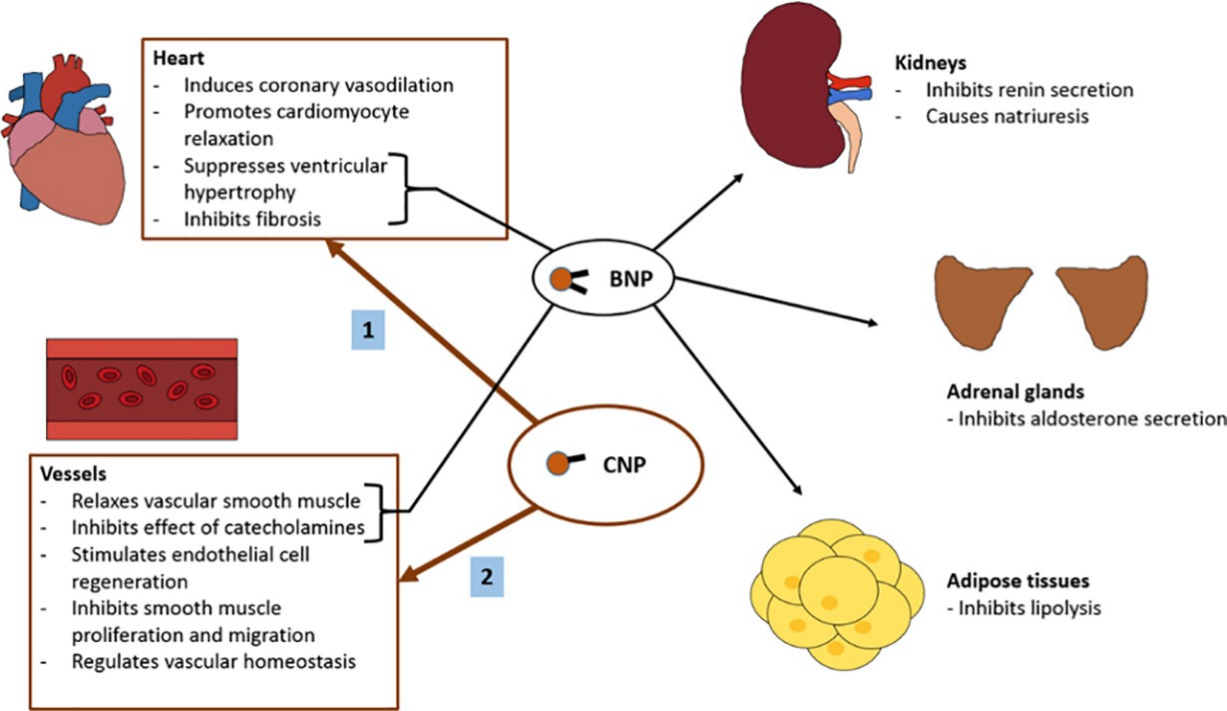
Function of C-type natriuretic peptide. CNP is an endothelium-derived peptide that is believed to have a cardioprotective function in both healthy and disease states. While BNP directly causes natriuresis in addition to other functions, CNP acts primarily on the heart and blood vessels. In the heart, CNP induces coronary vasodilation, promotes cardiomyocyte relaxation, and exerts antihypertrophic and antifibrotic effects. In the vessels, CNP relaxes smooth muscle and inhibits its proliferation and migration, while also inhibiting catecholaminergic effects, stimulating endothelial cell regeneration, and regulating vascular homeostasis. (BNP–B-type natriuretic peptide, CNP–C-type natriuretic peptide).

Our hypothesis-generating observations are interesting and deserve clarification from future mechanistic studies. However, we postulate that our observations may be explained by differences in functional characteristics between NTproBNP and NTproCNP. NTproBNP is primarily secreted by cardiomyocytes in response to increased ventricular stretching and serves a natriuretic function, particularly observed in heart failure states, where natriuretic function is critical in restoring fluid balance in heart failure. On the other hand, in non-heart failure states, NTproCNP may represent a ‘protective’ function, such as in cardioprotection, to maintain cardiovascular function in health[31,32]. CNP has been found to inhibit endothelin-1 induced cardiac myocyte hypertrophy via a cGMP-dependent mechanism, with antihypertrophic and antifibrotic effects on the heart[33]. Studies show that CNP fulfils this function via regulating vascular homeostasis, promoting cardiomyocyte relaxation, stimulating endothelial cell regeneration, inducing coronary vasodilation, inhibiting vascular smooth muscle proliferation and migration, and suppressing cardiac fibroblast proliferation[28,34–37] (Fig 1). Thus, lower levels of NTproCNP may represent lower levels of cardioprotection. The question as to whether NTproCNP is merely a bystander representing healthy myocardium or a key player involved in preservation/protection of myocardial function requires future mechanistic studies.

Further, in the evaluation of NTproCNP as a potential biomarker of myocardial function in ageing, our study attempted to determine a cut-off value of NTproCNP that could identify impaired myocardial relaxation with reasonable specificity. Larger studies in the future are necessary to confirm our observations about appropriate cut-offs for NTproCNP, adjusted to specificity or sensitivity thresholds. However, our data provides a glimpse of how NTproCNP levels may be handled in similar investigations.

We acknowledge limitations of our study. Firstly, NTproCNP levels are assumed to reflect levels of CNP. It is possible that NTproCNP and CNP levels do not correlate perfectly in milieu. However, prior reports have suggested significant correlations observed between NTproCNP and CNP such that NTproCNP could be used as a suitable substitute for assessment of CNP status in the circulation[19]. Secondly, our cross-sectional observations preclude causal inferences between NTproCNP and myocardial function. Longitudinal observations investigating changes in NTproCNP in relation to myocardial function in the same cohort, in addition to mechanistic studies, may provide stronger causal inferences in the future. Thirdly, our results are only applicable to elderly community adults, and results may differ in other community cohorts of different age and ethnicity. Fourth, we only studied one aspect of myocardial function, which is impaired myocardial relaxation. The association between NTproCNP may differ according to the type of cardiovascular dysfunction studied. Finally, while we observed statistically significant associations, the sample size was small and limited our ability to perform subgroup analyses.

In summary, our findings suggest that NTproCNP could be a suitable candidate biomarker for cardiac ageing, advancing our understanding of mechanisms involved in cardiac ageing.

## Conclusion

This study demonstrates the utility of NTproCNP as a potential biomarker of cardiac ageing. NTproCNP may be a promising circulating biomarker that can provide insights into myocardial ageing states, useful for future mechanistic and interventional studies.
